# Genetically Modified Mouse Models of Congenital Diaphragmatic Hernia: Opportunities and Limitations for Studying Altered Lung Development

**DOI:** 10.3389/fped.2022.867307

**Published:** 2022-05-13

**Authors:** Florian Friedmacher, Udo Rolle, Prem Puri

**Affiliations:** ^1^Department of Pediatric Surgery, University Hospital Frankfurt, Goethe University Frankfurt, Frankfurt, Germany; ^2^Beacon Hospital, University College Dublin, Dublin, Ireland; ^3^Conway Institute of Biomolecular and Biomedical Research, School of Medicine, University College Dublin, Dublin, Ireland

**Keywords:** congenital diaphragmatic hernia, diaphragm development, lung development, pulmonary hypoplasia, pulmonary hypertension, genetic model, transgenic mice, retinoic acid

## Abstract

Congenital diaphragmatic hernia (CDH) is a relatively common and life-threatening birth defect, characterized by an abnormal opening in the primordial diaphragm that interferes with normal lung development. As a result, CDH is accompanied by immature and hypoplastic lungs, being the leading cause of morbidity and mortality in patients with this condition. In recent decades, various animal models have contributed novel insights into the pathogenic mechanisms underlying CDH and associated pulmonary hypoplasia. In particular, the generation of genetically modified mouse models, which show both diaphragm and lung abnormalities, has resulted in the discovery of multiple genes and signaling pathways involved in the pathogenesis of CDH. This article aims to offer an up-to-date overview on CDH-implicated transcription factors, molecules regulating cell migration and signal transduction as well as components contributing to the formation of extracellular matrix, whilst also discussing the significance of these genetic models for studying altered lung development with regard to the human situation.

## Introduction

Congenital diaphragmatic hernia (CDH) represents a relatively common and life-threatening birth defect with an estimated global prevalence of 2.3 in 10,000 live births (1, 2). It is characterized by incomplete formation and/or muscularization of the primordial diaphragm, which allows herniation of abdominal viscera into the thoracic cavity, thereby filling space usually reserved to hold the growing lung (3, 4). Hence, pulmonary development is disrupted, leading to immature and hypoplastic lungs (5–7). Today, more than 70% of CDH cases are diagnosed prenatally based on maternal-fetal ultrasound or magnetic resonance imaging in the second trimester of pregnancy, thus potentially altering future outcome (8, 9). Depending on the extent of pulmonary hypoplasia, newborns with CDH often present with severe respiratory distress at birth, requiring immediate and complex treatment (10, 11). Although significant advances have been achieved in postnatal resuscitation and ventilation strategies over the past decades (12, 13), CDH continues to be one of the major challenges in neonatal intensive care with mortality rates ranging between 30 and 50% (14–16). Surgical repair of CDH is generally performed after clinical stabilization either by primary closure or in larger defects by reconstruction using a prosthetic patch or muscle flap (17, 18). While newer therapeutic measures such as gentle ventilation techniques, high-frequency oscillation and extracorporeal membrane oxygenation have improved overall survival rates (19–21), this has led to substantial long-term morbidity in CDH patients (22, 23), including chronic lung disease, gastroesophageal reflux, scoliosis, sensorineural hearing loss and neurodevelopmental deficits (24–26).

A defect in the posterolateral diaphragm (also referred to as Bochdalek hernia) is the most common type of CDH and comprises approximately 80–90% of all cases, with the majority being left-sided (85%), less often right-sided (10%), or bilaterally (<5%) (27). The dual-hit hypothesis explains CDH-associated pulmonary hypoplasia by an initial disruption in bilateral lung organogenesis before diaphragm closure, in combination with a second ipsilateral insult resulting from the intrathoracic herniation and subsequent restriction of fetal breathing movements (28). Typical features of pulmonary hypoplasia in CDH are structural immaturity and smaller lung volume with a significantly reduced number of terminal airways, disrupted alveologenesis, diminished alveolar airspaces, thickened alveolar walls accompanied by increased interstitial tissue and decreased gas-exchange surface area (29). These findings have indicated that the pulmonary anomalies in CDH are at least partially independent of the diaphragmatic defect, suggesting a potential developmental linkage between both organs at a molecular level. Much of our present knowledge on the morphogenetic lung abnormalities in CDH has derived from experimental animal research (30–33). Because diaphragm and pulmonary evolution is remarkably similar between mice and humans, mouse models represent a crucial aspect in advancing our insight into the pathogenic mechanisms underlying CDH and associated lung hypoplasia.

This article aims to offer a comprehensive overview of genetically modified mouse models of CDH, resultant candidate genes and signaling pathways, whilst also discussing new opportunities and limitations for studying altered lung development in relation to the human situation.

## Overview of Genetically Modified Mouse Models of Congenital Diaphragmatic Hernia

A large variety of genetic factors have been found to play key roles during the pathogenesis of CDH and pulmonary hypoplasia. Currently, genetic causes are detected in about 30% of CDH patients (34–36). Through recent advances in genetic engineering technologies, genetically modified mouse models of CDH are now frequently used in basic science research (37), offering several potential genes and signaling pathways involved in the etiology of diaphragmatic defects and allied lung anomalies (Table 1).

**TABLE 1:** Genetically modified mouse models of congenital diaphragmatic hernia.

Mouse models	Full gene names ^(functions)^	Diaphragmatic defects	Lung abnormalities
*ChAT^–/–^*	Choline actelytransferase^d^	Central hernia	–
*Chtop^–/–^* or *Chtop*^tr/tr^	Chromatin target of Prmt 1^a^	Posterolateral hernia	Hypoplasia
*DNase II*α*^–/–^*	Desoxyribonuclease IIα^d^	Malformed diaphragm with hernia	Lungs not inflated
*Eya1^–/–^;Eya2^–/+^*	Eyes absent homolog 1 and 2^a^	Absent diaphragm	Hypoplasia, reduced epithelial branching, increased mesenchmal cellularity
*Fbn1^–/–^*	Fibrillin 1^c^	Unspecified hernia with ruptured edges	–
*Fbln4^–/–^*	Fibulin 4^c^	Severe hernia with rupture	Defective distal airways, emphysema
Fras1^Q1263*/Q1263*^ ^†^	Fraser extracellular matrix complex, subunit 1^c^	Retrosternal hernia (with sac)	–
*Frem1^eyes2/eyes2^* ^†^	Fras1-related extracellular matrix 1^c^	Retrosternal hernia (with sac)	Long lobulation defects, fused pulmonary lobes
*Frem2*^ne/ne^ or *Frem2^b2b3270Clo^*^†^	Fras1-related extracellular matrix 2^c^	Retrosternal hernia (with sac)	–
*Fuz^b2b1273Clo^* ^†^	Fuzzy planar cell polarity protein^b^	Unspecified hernia	Hypoplasia, single left lung lobe
*Gata4^+/Δex2^*	GATA-binging protein 4^a^	Retrosternal hernia (with sac)	Dilated distal airways, increased saccule size, thickened mesenchyme, abnormal vasculature
*Gli2^–/–^, Gli3^–/–^* or *Gli2^–/–^;Gli3^+/–^*	GLI familiy zinc finger 2 and 3^a^	Posterior hernia	Hypoplasia, absent right accessory lobe, thickened mesenchyme
*Hlx^–/–^*	H2.0-like homeobox^a^	Muscular hypoplasia with unspecified hernia	Enlarged lungs with normal structure
*Hoxb4* ^PolII^	Homeobox B4^a^	Unspecified hernia	–
*Kif7*^dd/dd†^ or *Kif7*^maki†^	Kinesin family number 7^a, b^	Posterior hernia	Hypoplasia, reduced alveolar epithelial cell differentiation
*Lox^–/–^* or *Lox^b2b370^.^2Clo^*	Lysyl oxidase^c, d^	Central hernia with rupture	Hypoplasia, abnormal acini, abnormal elastic fibers
*Lrp1^b2b1554Clo^* ^†^	Low density lipoprotein receptor-related protein 1^b, d^	Unspecified hernia	–
*Met^–/–^*	Mesenchymal-epithelial transition factor^b^	Amascular diaphragm with hernia	Abnormal saccule morphology
*Mtpa^–/–^*	Mitochondrial trifunctional enzyme α^d^	Unspecified lesions	–
*MyoR^–/–^* (*Msc^–/–^*); *Cap^–/–^* (*Tcf21^–/–^*)	Myogenic bHLH transcription factor R (Musculin)^a^; Transcription factor 21 (Capsulin)^a^	Posterior hernia	Hypoplasia, defective branching morphogenesis, abscence of alveoli, abnormal vasculature
*Ndst1* ^ECKO^	*N*-deacetylase-*N*-sulfotransferase 1^b^	Central hernia	Thick interalveolar septa
*Nr2f2^–/–^* (*Couptf2^–/–^*)	Nuclear receptor subfamily 2, group F, number 2 (Chicken ovalbumin upstream promoter transcription factor 2)^a^	Posterolateral hernia	Hypoplasia
*Pbx1^–/–^*	Pre-B-cell leukemia transcription factor 1^a, b^	Muscularization and tissue patterning defect	Hypoplasia, alveolar simplification
*Pdgfr*α*^–/–^*	Platelet-derived growth factor receptor, α-polypeptide^b^	Posterolateral hernia	Hypoplasia, failure of alveogenesis
*Pls3^W499C^*	Plastin 3^b, c^	Posterolateral and anterior muscular thinning, hernia	–
*Rar*α*^–/–^;Rar*β*^–/–^*	Retinoic acid receptor α and β^a^	Posterior hernia	Hypoplasia, abnormal alveoli, lung agenesis
*Robo1^–/–^;Robo2^–/–^* or *Dutt1;Robo1^–/–^*	Roundabout guidance receptor 1 and 2^b^	Posterior hernia	Irregular bronchioles, reduced terminal air spaces, abnormal alveoli, thick septa
*Six1^–/–^*	Six homeobox 1^a, b^	Absent diaphragm	Hypoplasia, reduced branching morphogenesis, narrow bronchi, arrested expansion of epithelial tubules, dense mesenchymal cellularity, failure of lung maturation
*Slit3^–/–^*	Slit guidance ligand 3^b^	Central hernia (with sac)	–
*Sox7^+/Δex2^*	Sex determining region Y-box 7^a^	Retrosternal hernia (with sac)	–
*Wdr35* ^yet/yet†^	WD repeat domain 35^d^	Unspecified hernia	Hypoplasia
*Wt1*^–/–^ or conditinal knockouts (e.g., *Wt1^CreERT2/+^*;*Bcat*^fx^ or G2-Gata4^Cre^;Wt1^fl/fl^)	Wilms tumor 1^a^;β-catenin^a^	Posterolateral hernia	Hypoplasia, abnormally fused and malformed lung lobes, collapsed distal air spaces
*Zfpm2^–/–^* (*Fog2^–/–^*)^†^	Zinc finger protein, multitype 2 (Friend of GATA-binding protein 2)^a^	Posterolateral hernia (with sac)	Hypoplasia, absent right middle and accessory lobe

*^†^Chemically induced by N-ethyl-N-nitrosourea. ^a^Transcription factor or transcriptional (co-)activator. ^b^Cell migration, proliferation or mesodermal patterning. ^c^Formation of extracellular matrix. ^d^Signal transduction or apoptosis.*

## Transcription Factors and Transcriptional (Co-)Activators

Numerous transcription factors and transcriptional (co-)activators have been suggested in the development of the primordial diaphragm and lungs. Many of them are associated with retinoid or sonic hedgehog signaling pathways.

### Retinoid Signaling Pathway

Vitamin A (i.e., retinol) and its derivates (i.e., retinoids) are indispensable for various aspects of early embryogenesis. Over the years, several knockout models have indicated a role of the retinoid signaling pathway and its downstream targets in the pathogenesis of CDH (38). For instance, mice lacking both subtypes of *retinoic acid receptors* α and β (*Rar*α and *Rar*β) have been demonstrated to generate offspring with posterolateral diaphragmatic defects identical to those observed in human patients (39–44), and similar to the vitamin A-deficient CDH mouse model as previously reported by Anderson (45, 46). Surprisingly, single *Rar* null mutant mice did not display any of the predicted malformations that were seen in rats with vitamin A deficiency (31). Nevertheless, when the activity of several receptors was inhibited, various deformities were noted including right-sided CDH in *Rar*α/β*2* and left-sided CDH in *Rar*α/β*2*^+/–^ animals. Moreover, these mice exhibited severe pulmonary hypoplasia (31). Despite the convincing data, these genetically modified mouse models manifest only a comparatively low incidence of diaphragmatic defects and a high rate of additional comorbidities (e.g., cranial, cardiac, vertebral and limb), which do not accurately depict the human situation (39, 42). Still, mutations in the *stimulated by retinoic acid gene 6 (STRA6)*, a membrane receptor that controls the cellular uptake of vitamin A and *cellular retinoic acid binding protein 1 (CRABP1)*, which is located on chromosome 15, have been found to lead to a spectrum of developmental anomalies including CDH and hyperplastic lungs (47, 48).

### Nr2f2 (Couptf2)

Another important gene that is linked with the retinoid signaling pathway is *chick ovalbumin upstream promoter transcription factor II (COUP-TFII)*, a transcription factor that is affiliated with the nuclear steroid/thyroid hormone receptor superfamily, whose DNA-binding site has been shown to reduce the induction of retinoic acid receptors (49–51). *COUP-TFII* was recently renamed as *nuclear receptor subfamily 2 group F (NR2F2)*, which is expressed in the diaphragm and lungs during early gestation (34). Mapped to chromosome 15q26 in humans, the *NR2F2* gene is situated on a recognized CDH hotspot region, thus making it a likely contributor to the etiology of diaphragmatic defects. On the basis of this observation, You et al. (52) have created a tissue-specific *Nr2f2^–/–^* mouse model that features left-sided Bochdalek-type CDH and pulmonary hypoplasia similar to the human situation. Through targeted ablation of *Nr2f2* in the foregut mesenchyme and pleuroperitoneal folds (PPFs), posterolateral diaphragmatic defects presumably arise because of the failure of the posthepatic mesenchymal plate to merge with the lateral body wall, thus enabling stomach and liver to protrude into the chest (52).

### Wt1

The creation of genetic mouse models for various other applications has revealed several genes, which one would not necessarily immediately associate with CDH. Initially introduced as a model for the investigation of early urogenital organogenesis (53), *Wilm’s tumor 1 (Wt1)* null mutant mice die during mid-gestation, displaying posterolateral diaphragmatic defects and lung hypoplasia alongside urogenital abnormalities (54–56). Heterozygous mutations of the *WT1* gene, which encodes a transcription factor that contains four zinc finger motifs, is known to produce distinct syndromes with clinical overlap that include CDH (e.g., Denys-Drash syndrome or Meacham syndrome) (57, 58). *Wt1^–/–^*, vitamin A-deficient and nitrofen mouse models of CDH each implied a mutual pathomechanism for the formation of diaphragmatic defects with several analogies to the condition in humans (59). More recently, Carmona et al. (60) and Cleal et al. (61) have reported that conditional deletion of *Wt1* in the mesenchyme of the septum transversum can cause CDH in mice. Today, it is proven that *Wt1* and *Couptf2* both interact with the retinoid signaling pathway during embryonic development (3). Surprisingly, *Wt1* and *Couptf2* are not found in the muscle precursors but in the non-muscular mesenchymal compartment of the PPFs (3). Paris et al. (62) have developed a novel genetically modified mouse model of CDH, demonstrating that *Wt1*-induced β-catenin loss-of-function produces posterior diaphragmatic defects, bilateral pulmonary hypoplasia and liver herniation, comparable to the phenotypes associated with CDH in human patients. Additionally, a decreased mesothelial proliferation and increased rate of cell death was identified in the posterior diaphragm mesenchyme, and all mouse pups died postnatally with malformed lung lobes and collapsed distal air spaces (62). Loss of *Wt1* has also been associated with lung branching defects before diaphragm closure in another genetic model of CDH (63).

### Sonic Hedgehog Signaling Pathway

*GLI-Kruppel family member 2 (Gli2)* and *Gli3* and are both members of a highly conserved morphogenetic family, belonging to the sonic hedgehog (Shh) signaling pathway (64). This pathway is thought to be crucial during normal diaphragmatic development (36). A murine model of the VACTERL-like syndrome (i.e., vertebral, anorectal, cardiac, tracheoesophageal, renal and limb anomalies) created by Kim et al. (65) involved *Gli2^–/–^*;*Gli3^–/–^* and *Gli2^–/–^*;*Gli3*^+/–^ mice that developed left-sided posterior CDH and pulmonary hypoplasia besides the observed VACTERL components. This was the first experimental model that reproduced the human VACTERL association, indicating that disruptions in *Shh* signaling might contribute to the pathogenesis of VACTERL syndrome. Likewise, as *Gli2*, *Gli3* and *Wt1* all encode important zinc finger proteins, further transcription factors of this type have been hypothesized through the generation of newer genetic animal models of CDH. For example, *kinesin family member 7 (Kif7)* and *pre-B-cell leukemia transcription factor 1 (Pbx1)* were recently recognized as indispensable components of the *Shh* signaling pathway, functioning as regulators during early embryogenesis (66, 67). *Kif7* encodes a motor protein that functions downstream of the transmembrane receptor *smoothened*, and interacts with both *Gli2* and *Gli3* (68). Furthermore, *Kif7* was found to coordinate cell proliferation, central tendon patterning and differentiation of the primordial diaphragm in a genetically modified mouse model of CDH (69). Homozygous *Kif7^dd/dd^* mutant mice and *Pbx1^–/–^* knockout mice both display left-sided posterior diaphragmatic defects and hypoplastic lungs (36, 69–71). In turn, haploinsufficieny of *PBX1* has been associated with various congenital anomalies including CDH (72). Moreover, two predicted variants in the *KIF7* gene were recently detected in patients with CDH (73). Additionally, mice lacking *chromatin target of protein arginine methyltransferase 1 (Chtop)* have numerous developmental abnormalities including posterolateral defects in the diaphragm, pulmonary hypoplasia and liver herniation (74–76). High-resolution 3D imaging further characterized these diaphragmatic defects in *Chtop^–/–^* mice embryos (77).

### Zfpm2 (Fog2), Gata4 and Sox7

*Zinc finger protein 2 (ZFPM2)*, formerly known as *friend of GATA-binding protein 2 (FOG2)*, encodes another zinc finger-containing protein that regulates the transcriptional activity of *GATA4*, hereby controlling a number of developmental mechanisms in the forming diaphragm and lung (78–81). In mice, *Fog2* was initially found to be expressed in the embryonic septum transversum of the diaphragm (81). In humans, *ZFPM2* is located on chromosome 8p23 and has been demonstrated to interact with *COUP-TFII* (82, 83). However, only a single mutation in the *ZFPM2* gene has been identified in isolated patients with non-syndromic CDH to date (31). In a cohort of 275 patients with CDH, Longoni et al. (84) have recently reported the incidence of *ZFPM2* mutations to be nearly 5%. In addition, their genetic analysis of a multigenerational family revealed a heritable intragenic *ZFPM2* deletion with an approximated penetrance for clinical relevant diaphragmatic defects of around 37.5% (84). On the other side, mice exposed to the chemical mutagen *N*-ethyl-*N*-nitrosourea (ENU) generated *Fog2*^–/^*^–^* offspring with bilateral hypoplastic lungs and a defective posterolateral diaphragm characteristic of CDH (80), while 70% of mice heterozygous for a *Gata4* deletion mutation (i.e., *Gata4^+/Δ*ex*2^*) displayed retrosternal diaphragmatic defects, dilated distal airways and thickened pulmonary mesenchyme (85). Using genetically modified mice, Merrell et al. (86) have shown that *Gata4* mosaic mutations in PPF-derived muscle connective tissue fibroblasts led to the development of localized amuscular regions of the diaphragm, which were biomechanically weaker and subsequently caused CDH. *GATA4* and *ZFPM2* genes have been both found to be absent in humans with CDH (78), emphasizing their roles as possible candidate genes for CDH. Moreover, *Zfpm2* is known to interact with *Nr2f2*, indicating that these two transcription factors together with *Gata4* may contribute to diaphragm formation (83). Recurrent microdeletions of 8p23.1, including *GATA4* and the *sex determining region Y-box 7 (SOX7)* gene are accompanied with a significant risk of CDH and cardiovascular anomalies (87). Even though mice lacking the *Gata4* gene display both diaphragmatic and cardiac defects, no human patient with cardiac anomalies and *GATA4* mutations have been identified with CDH so far (87). However, Wat et al. (87) have recently demonstrated that haploinsufficiency of *Sox7* or *Gata4* is enough to cause retrosternal diaphragmatic defects in mice and that haploinsufficiency of *SOX7* and *GATA4* may in turn be involved in the pathogenesis of CDH in patients with 8p23.1 deletions.

### Cap (Tcf21) and MyoR (Msc)

Several basic helix-loop-helix transcription factors have been shown to support the development of the primordial diaphragm in mice. *Capsulin (Cap)* is one of those, which is strongly expressed in the fetal diaphragm and in mesenchymal cells of the lung (88). As one might expect, *Cap*^+/–^ mice have severe defects in lung morphogenesis and lack alveoli (89). On the other hand, mice homozygous deficient for both *cap^–/–^* and the related *myogenic bHLH transcription factor R (MyoR^–/–^)* lack facial musculature and exhibit posterior diaphragmatic defects. This double mutant mouse model, which was generated at first to study the formation of facial muscles, displayed not only CDH and defective lung branching morphogenesis but also severe facial muscle abnormalities (90). Although these genetically modified mice died soon after birth because of pulmonary and cardiac malformations, the type of diaphragmatic defect seen in this model indicates that both *Cap* and *MyoR* are necessary for the integrity of the developing diaphragm. Previously, these genes were referred to as *transcription factor 21 (Tcf21)* and *musculin (Msc)*, respectively (90, 91).

### Eya1 and Six1

*Eyes absent (Eya)* genes and the transcription factor *sine oculis homebox 1 (Six1)* form an important signaling network, which plays a central role during embryonic development (92, 93). *Eya1* and *Six1* together constitute an evolutionary conserved transcriptional complex that coordinates multiple integrated processes needed for normal growth of the primordial diaphragm and lung (94). Further research work has confirmed that the *Eya1-Six1* pathway has a key role in lung maturation by regulating its branching morphogenesis (95). Mice deficient in *Eya1^–^*^/^*^–^* and *Eya2^–^*^/+^ have no diaphragm (94), whereas single mutant *Eya1* mice die shortly after birth due to respiratory failure, having severely hypoplastic lungs with reduced epithelial branching and increased mesenchymal cellularity (95). In *Six1^–/–^* mice, the diaphragm is also absent, and the *Six1* deletion causes pulmonary hypoplasia with greatly reduced epithelial branching, narrow bronchi, dense mesenchyme and obvious failure of normal lung maturation (96, 97). These findings indicate that disruption of the *Eya1-Six1* signaling pathway may lead to neonatal lethality as a consequence of an absent diaphragm and hypoplastic lungs.

### Hlx and Hoxb4

*H2.0-like homeobox (Hlx)* is a protein coding gene that is relatively conserved across various species (98). This homeobox transcription factor has been found to be highly expressed during early organogenesis in the septum transversum of the diaphragm and lung mesenchyme (99, 100). *Hlx^–/–^* mice suffered early demise and showed diaphragmatic defects (101). Additionally, Farrell et al. (102) reported two human fetuses with multiple congenital anomalies including CDH that were homozygous for a missense variant in the *HLX* gene. The *Hox* gene family encodes for multiple transcription factors that have crucial regulatory functions during embryonic development (103). Targeted mutation of the *homeobox B4 (Hoxb4)* gene in mice resulted in offspring with poorly formed diaphragms and diaphragmatic defects, strikingly similar to the phenotype seen in humans with anterior CDH (104).

## Molecules Implicated in Cell Migration, Proliferation and Mesodermal Patterning

Various genes and enzymes involved in cell migration, proliferation and mesodermal patterning have been found to be associated with embryonic diaphragm and lung development.

### Slit3, Robo1/2, Ndst1 and Pdgfra

The Slit guidance ligand (Slit) family of proteins comprise a group of molecules with crucial functions in cell migration and adhesion through interaction with roundabout (Robo) receptors. *Slit* genes are expressed in the mesothelium of the diaphragm during embryogenesis (105). Homozygous *Slit3^–/–^* mice experience faulty detachment of the central tendon region of the diaphragm from the underlying liver due to connective tissue defects, thus causing central-type (i.e., septum transversum) CDH (105, 106). Therefore, this genetically modified model is facing the disadvantage of having the diaphragmatic defect on or near the ventral midline portion of the central tendon as opposed to the posterolateral diaphragm, thus representing less than 5% of CDH cases seen in human patients. Further malformations in this mouse model include ureteric and renal agenesis in combination with intrathoracic herniation of liver and gallbladder (106), which again occurs infrequently in humans with CDH. Until now, no *SLIT3* mutations have been identified in CDH patients. *Robo* genes encode large transmembrane receptors that are involved together with their ligands in numerus developmental mechanisms (107–109). For example, the *Slit*-*Robo* signaling pathway has been reported to have various fundamental functions including axon guidance, neural crest cell migration, epithelial cell adhesion, embryonic heart formation as well as diaphragm and kidney development (105–107, 110–114). Inactivation of *Robo1* and *Robo2* genes in mice has been shown to cause diaphragmatic defects and subsequent herniation of the stomach into the thorax, which leads to poor lung inflation and perinatal death, similar to human CDH cases (107). Homozygous mice with targeted deletion in the *Dutt1/Robo1* gene often die at birth due to respiratory failure, demonstrating delayed lung maturation and diaphragmatic defects in some instances (115). More recent studies identified the heparan sulfate proteoglycan as an essential part of the *Slit-Robo* signaling complex, which stabilizes the *Slit-Robo* interaction (116). Furthermore, Zhang et al. (117) have noted that absence of the heparan sulfate biosynthetic enzyme *N*-deacetylase-*N*-sulfotransferase-1 (Ndst1) in the mouse endothelium interferes with vascular development in the primordial diaphragm, resulting in hypoxia as well as diaphragmatic hypoplasia and central-type CDH. The observed phenotypes in these animals mirror the congenital anomalies seen in *Slit3* knockout mice. In addition, implementation of a heterozygous mutation in the *Robo4* gene, which encodes the receptor of Slit3, exacerbated the defect in vascular and diaphragmatic formation (117). Thus, these findings suggest that loss of *Ndst1* may lead to abnormal vasculogenesis in the diaphragm and CDH and that heparan sulfate in turn promotes the angiogenic *Slit3*-*Robo4* signaling cascade during normal vascular patterning. Apart from this, mice homozygous for null mutations in the *platelet-derived growth factor receptor* α *(Pdgfra)* gene exhibit not only posterolateral diaphragmatic defects, they also develop a spectrum of other comorbidities including cardiovascular anomalies, renal and urogenital malformations, facial clefts, lung hypoplasia and failure of alveogenesis (118, 119).

### Fuz, Met and Pls3

Inbred C57BL/6J mice chemically mutagenized with ENU displayed a previously unknown mutation in the *fuzzy planar cell polarity protein (Fuz)* that was associated with CDH, liver protrusion into the chest cavity and pulmonary hypoplasia with a single left lung lobe (120). The *mesenchymal-epithelial transition factor (Met)* gene encodes for a receptor tyrosine kinase that is necessary for the migration of muscle precursor cells into the forming diaphragm (121), whereas *fibroblast growth factor 10 (Fgf10)* is crucial for early organogenesis of the lung (122). Oral administration of the herbicide nitrofen in *Met^–/–^* mice with amuscular diaphragms and *Fgf10^–/–^* mice with hypoplastic lungs resulted in CDH in both murine models, indicating that diaphragmatic defects may develop independently of myogenesis and pulmonary development (123). A novel missense variant affecting the actin-binding domains of *plastin 3 (PLS3)* was recently identified in eight unrelated families, causing X-linked CDH and body wall defects. A genetically modified mouse model of this *Pls3^W499C^* variant resulted in perinatal death and reproduced the main features of the human phenotype, including diaphragmatic and body wall abnormalities (124). An abnormal plastin-actin interaction is the most likely explanation for the observed congenital malformation in both humans and mice.

## Components Involved in the Formation of Extracellular Matrix

Normal development of the primordial diaphragm and lung is also dependent on the proper formation of its underlying extracellular matrix (ECM). Today, several components of the ECM are known to be aberrant in CDH and associated lung defects.

Fraser extracellular matrix complex subunit 1 (Fras1), Fras1-related extracellular matrix 1 (Frem1) and Frem2 form a mutually stabilizing ternary complex in the ECM, which plays a critical role in cell adhesion and intercellular signaling (125, 126). After identification of a novel *FREM1* deletion in a female infant with isolated left-sided CDH and a membranous sac, Beck et al. (127) developed a *Frem1*-deficient mouse model that displays a comparable phenotype with retrosternal diaphragmatic defect and reduced levels of cell proliferation in the anterior portion of the growing diaphragm, hereby showing that a deficit of *FREM1* can lead to CDH in both humans and mice. Because of the observed phenotypic similarities between *Frem1*-deficient mice and mice lacking the retinoic acid-responsive transcription factor *Gata4*, the same author group conducted further studies, revealing that *Frem1* interacts not only with *Gata4* but also with *Slit3* in this mouse model of CDH and concomitant lung lobulation defects (128). More recently, Jordan et al. (129) reported that *Frem2^ne/ne^* and *Fras1*^*Q*1263*/*Q*1263*^ mice developed an almost identical type of anterior midline CDH with herniated viscera covered by a thin membranous sac as seen in *Frem1*-defcient mice, thus concluding that loss of the *Frem1/Frem2/Fras1* complex or its function results in retrosternal CDH in these animals. The cross-linking of collagens and elastin, which is essential for the structural stability of the ECM, is catalyzed by lysyl oxidase (Lox), an extracellular cuproenzyme (130). In turn, *Lox^–/–^* mice die at birth, having a ruptured diaphragm as a result of fragmentation in the central tendon (131, 132). However, no human *LOX* mutations have been reported so far. Another ECM protein associated with the pathogenesis of CDH is fibrillin 1 (Fbn1), an integral part of microfibrils in elastic and non-elastic connective tissues (133). A gene-targeting mutation of the mouse *Fbn1* gene has been associated with diaphragmatic defects and histological examination revealed a focal inflammatory infiltrate at the ruptured edges (134). These homozygous *Fbn1^–/–^* mutant mice died postnatally due to pulmonary insufficiency, exhibiting CDH and herniation of abdominal viscera into the thoracic cavity (134). Fibulin 4 (Fbln4) also belongs to a family of ECM proteins, which controls fiber assembly and is known to bind Lox (135). *Fbln4* null mutant mice die just after birth with severe CDH and rupture of the diaphragm, in addition to defective distal airways and lung emphysema (136).

## Additional Genes and Enzymes Participating in Signal Transduction and Apoptosis

Several other genes and enzymes responsible for signal transduction, intracellular signaling and apoptosis have been discovered in association with diaphragmatic defects in genetically modified mouse models.

The acetylcholine-synthesizing enzyme choline acetyltransferase (ChAT) has been reported to be implicated in various morphogenetic processes during embryonic development (137, 138). In fact, cross-sections of the diaphragm from *ChAT^–/–^* mice showed liver herniation through the tendinous center of the diaphragm (139), presumably as a consequence of impaired muscle formation. Deoxyribonuclease IIα (DNase IIα) belongs to a large group of endonucleases involved in DNA digestion during apoptosis. *DNase II*α*^–/–^* mice displayed a malformed diaphragm with hernia and non-inflated lungs, suggesting that these animals suffer perinatal lethality because of a dysfunctional diaphragm and associated respiratory insufficiency (140). Low-density lipoprotein receptor-related protein 1 (Lrp1) is crucial for proper embryonic development through regulation of intracellular signaling cascades (141). ENU-induced mutation in the *Lrp1* gene of mice resulted in body wall closure defects with CDH and liver protruding outside of the abdominal cavity (142). Mitochondrial trifunctional protein (Mtp) is a multi-enzyme complex of four α and four β subunits that catalyzes oxidation of long-chain fatty acids, which is essential for normal embryogenesis (143). *Mtp*α*^–/–^* knockout mice suffer neonatal death with cardiac and diaphragmatic defects, indicating that deficiency of *Mtpa* may cause dysfunction of the diaphragm and subsequent respiratory insufficiency (143). WD repeat domain 35 (Wdr35) is a protein coding gene, which participates in intracellular trafficking, cargo recognition and binding during embryonic development (144). Following a recessive ENU mutagenesis screen for genes affecting embryogenesis, Mill et al. (144) noticed that mutant *Wdr35^yet/yet^* mice embryos died before birth, displaying diaphragmatic defects and hypoplastic lungs.

## Conclusion and Future Directions

Over the years, experimental animal models of CDH have not only permitted us to investigate the pathogenesis of this relatively common but complex birth defect in more detail, they have also led to a better understanding of the molecular genetic basis of the underlying tissue defects. Therefore, animals with CDH in which this congenital anomaly develops naturally represent the ideal research models to study disease pathomechanisms and related lung abnormalities, as there is minimal interference to the animal before the examination. Furthermore, genetically modified animal models of CDH not only resemble the natural development of this malformation, they also provide new insights into the participating genes and signaling pathways, and how their modification can potentially change the course of this life-threatening condition. With the recent advent of novel molecular techniques including biomedical engineering and ENU mutagenesis screens, we hopefully may identify additional CDH-related mutations that are linked with abnormal diaphragm and lung development in other genetic mouse models (145–147).

## Author Contributions

FF, UR, and PP critically revised the initial manuscript draft for important intellectual content and performed the literature search for the work. FF outlined and wrote the initial manuscript draft. All authors approved the final version to be published and agree to be accountable for all aspects of the work in ensuring that questions related to the accuracy or integrity of any part of the work are appropriately investigated and resolved.

## Conflict of Interest

The authors declare that the research was conducted in the absence of any commercial or financial relationships that could be construed as a potential conflict of interest.

## Publisher’s Note

All claims expressed in this article are solely those of the authors and do not necessarily represent those of their affiliated organizations, or those of the publisher, the editors and the reviewers. Any product that may be evaluated in this article, or claim that may be made by its manufacturer, is not guaranteed or endorsed by the publisher.
